# The ROSA knee robotic system demonstrates superior precision in restoring joint line height and posterior condylar offset compared to conventional manual TKA: a retrospective case–control study

**DOI:** 10.1007/s00590-024-03942-6

**Published:** 2024-04-18

**Authors:** Zakareya Gamie, George Paparoidamis, Nikos Milonakis, Eustathios Kenanidis, Eleftherios Tsiridis

**Affiliations:** 1 Tsiridis Orthopaedic Institute, ICAROS Clinic, Thessaloniki, Greece; 2 Academic Orthopaedic Department, Papageorgiou General Hospital, Aristotle University of Thessaloniki, Thessaloniki, Greece; 3 Centre of Orthopaedic and Regenerative Medicine (CORE), Center for Interdisciplinary Research and Innovation (CIRI), Aristotle University of Thessaloniki, Thessaloniki, Greece

**Keywords:** Knee, Joint, Osteoarthritis, Arthroplasty, Robotics, Technology, Restoration

## Abstract

**Purpose:**

Variations in total knee arthroplasty (TKA) joint line height may lead to complications such as pain and altered joint mechanics, while posterior condylar offset (PCO) can influence knee stability.

**Methods:**

Single-centre, single-surgeon retrospective analysis from December 2019 to May 2023 investigated primary unilateral TKA (Nexgen Legacy, Zimmer Biomet) in patients with knee osteoarthritis, using ROSA robotic system (raTKA) or conventional manual technique (mTKA). Joint line height and PCO were measured and compared in 182 raTKA and 144 mTKA patients.

**Results:**

The groups were matched in age (*p* = 0.847) and sex distribution (*p* = 0.2). Excellent interobserver agreement (ICC ≥ 0.9). RaTKA mean joint line height difference was − 0.0001 mm (± 3.48, 95% CI − 0.509, 0.509) (*p* = 0.523), − 0.951 mm for mTKA (± 4.33, 95% CI − 1.664, − 0.237) (*p* = 0.009). RaTKA mean PCO difference was 0.52 mm (± 2.45, 95% CI 0.160, 0.880) (*p* = 0.005), 1.15 mm for mTKA (± 4.01, 95% CI – 1.496, 1.818) (*p* < 0.001). Mean difference in joint line height of 0.95 mm between groups was significant (*p* = 0.027), and for PCO, it was 0.63 mm, demonstrating tendency towards significance (*p* = 0.08). Mean absolute value in joint line height difference between groups was not significant (*p* = 0.235) but highly significant for PCO (*p* < 0.001).

**Conclusion:**

The ROSA knee robotic system can more accurately restore joint line height and PCO compared to conventional manual TKA. The improved degree of precision raTKA offers may be a vehicle for better Patient-Reported Outcome Measures, but further correlational studies are required.

## Introduction

Joint line elevation following total knee arthroplasty (TKA) can have negative effects such as impingement and reduced range of movement (ROM) [1–3]. Distal displacement can also result in pain and subluxation [4]. Early investigations report a joint line elevation greater than 8 mm as concerning [5–7]. Patellofemoral joint contact forces can increase by 60% when elevation is 10 mm in revision knee arthroplasty, where anatomical landmarks are difficult to palpate [8], restoration of which improves the patellar score [9]. More recently, in primary knee arthroplasty, there can be deterioration of functional scores with ≥ 5 mm of elevation [2] and no significant effect when it is in the region of − 1 mm to 5 mm compared to the preoperative value [10]. A systematic review has also found that exceeding more than 4 mm is associated with statistically significant lower outcome scores [11].

Various measurement techniques have been developed and utilized to calculate the differences in joint line pre- and post-operatively [12, 13]. The NAVIO robotic system, which utilizes a saw instead of a burr, has recently been compared to the conventional manual technique. Joint line height change was assessed with the Imperial Joint Line Congruency Measurement (IJLCM) method and found to be significantly better when using the robotic system, with an average decrease of − 0.38 mm compared to 0.91 mm of elevation with the conventional manual technique. The study also investigated the change in posterior condylar offset (PCO) and found it to be more improved with the NAVIO robotic system, with a change of 0.08 mm vs 1.64 mm for the manual technique compared to the preoperative value. There is currently a requirement to build up evidence for the individual robotic systems [14]; therefore, we undertook a study to assess joint line height and PCO change specifically for the ROSA robotic system (Zimmer Biomet, Warsaw, IN) in comparison with the manual technique in cases performed by a senior surgeon using the same implant.

## Methods

### Study design & patient selection

This study took place at an academic orthopaedic institution and was approved by the Hospital Health Research Ethics Board. Patients provided informed consent, and the data evaluated during the study were collected from the regional academic Arthroplasty Registry Thessaloniki (ART).

A single-centre, single senior surgeon retrospective comparative analysis from December 2019 to May 2023 was carried out investigating primary unilateral TKA in patients with knee osteoarthritis, performed using either the ROSA robotic system (raTKA) or the conventional manual jig-based technique (mTKA) utilizing the same prosthesis (Nexgen Legacy, Zimmer Biomet, Warsaw, IN). The two groups were matched for age and sex using frequency matching and the surgeon performed robotic procedures that were outside of his learning curve.

### Inclusion and exclusion criteria

Patients that had knee OA were included in the study. The exclusion criteria were patients who underwent complex primary or revision TKA and those who were implanted with a different knee implant. Furthermore, patients were excluded if there was a significant preoperative fixed flexion deformity, if the anatomical landmarks used were not visible in radiographs and if the longitudinal axes could not be determined.

### Radiographic quality and measurement

As part of our unit’s protocol, all patients had weight-bearing knee X-rays pre- and postoperatively. Joint line (JL) height was assessed with the “IJLCM” technique, with the formula as ‘[(joint line height post-op (mm)—joint line height pre-op (mm)]’ [13]. Posterior condylar offset (PCO) was also assessed using the method described by Bellemans et al*.,* involving the formula ‘[(posterior condylar offset post-op (mm)—posterior condylar offset pre-op (mm)]’ [15]. Measurements were undertaken by two observers, and the mean value was used for statistical analysis.

### Surgical technique

Fully cemented posterior stabilized TKAs were performed on all patients, with tourniquet applied and drain used for the first day following surgery. We aimed to restore the inherent bony alignment and balance the soft tissues’ laxity and place implants in a manner tailored to the patient’s anatomy and soft tissue laxity within defined limits. The surgeon first performed the necessary soft tissue releases and then the bone resections to achieve balanced flexion–extension gaps and equal mediolateral soft tissue tension. The bone resections were adjusted within the limits of ± 5^°^ varus-valgus cuts of the ROSA system. To achieve symmetrical and balanced gaps for the manually performed cases, we used ≤ 2 mm femoral or tibial varus/valgus cuts.

TKA using the ROSA robotic system (Zimmer Biomet, Warsaw, IN) followed a standard protocol. After a medial parapatellar approach, arrays were placed into the tibia and femur to track the patient’s tibia and femur. All necessary soft tissue releases were then performed. Digitization of the landmarks was undertaken, followed by an assessment of the alignment and laxity profile. Parameters such as component and gap size were then demonstrated on the panels. The extension gap was first evaluated. The distal femur and proximal tibia resections were undertaken after the selection of the desired resection thickness and angle, followed by the validation of the bone cuts. The flexion gap distance was demonstrated following the femoral rotation assessment. Intraoperative validation of laxity was undertaken, followed by the completion of the femoral bone cuts. Trial components were applied, followed by an assessment of stability. If satisfactory, the components (NexGen Complete Knee Solution—LPS FLEX Posterior Stabilized) were then cemented.

For manual primary TKA, a standardized approach was followed. Following a medial parapatellar approach, all necessary soft tissue releases were performed. A 5° to 7° valgus angle cut was used for the distal femur, and the proximal tibia was cut perpendicular to the mechanical axis with a extramedullary jig. The rotation of the femoral implant was determined using the posterior condyle reference and balancing of flexion and extension gaps with additional soft tissue releases. Trial components were applied, followed by an assessment of stability and patella tracking. If satisfactory, the components were cemented in position.

### Statistical analysis

Using Lehr’s formula and based on previous studies [16], our statistical analysis found that with sufficient power of 0.8 and the *α* value of 0.05, to find significance for a joint line height and PCO difference of 0.5 mm (CI = 0.8 mm) between the ROSA robotic system and manual TKA, at least 41 patients in each group had to be enrolled.

Normality assumptions were evaluated using the Kolmogorov–Smirnov or Shapiro–Wilk test. Levene’s test was used to assess the homogeneity of variances for all variables. For comparison of the age distributions between the two groups, a Mann–Whitney test was employed. The sex and operation side, across the groups, was assessed using Pearson chi-squared tests. For the examination of within-group differences in joint line and PCO measurements pre- and postoperatively, we used Wilcoxon signed-rank tests and paired *t* tests, taking into consideration the normality of the data and the paired nature of observations. Between-group differences in joint line and PCO measurements were investigated using independent *t* tests.

Inter-observer agreement was quantified using intra-class correlation (ICC), providing insights into the consistency of observations between the two observers. Statistical tests were conducted using the R software (RStudio Integrated Development Environment for R, Boston, Massachusetts.), with significance deemed at a *p* value < 0.05.

## Results

**Fig. 1:**
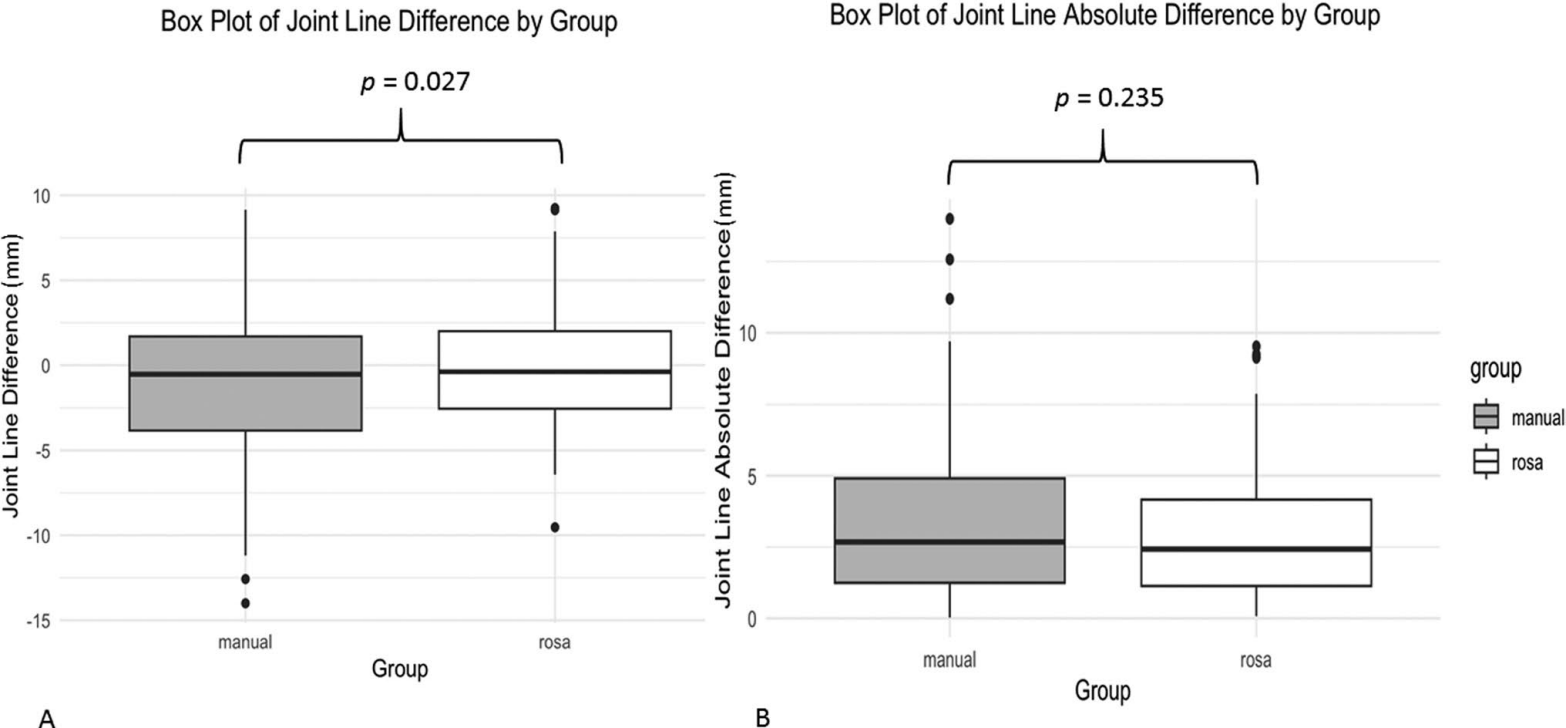
**A** Box plot of Joint Line Height average difference by group raTKA vs mTKA. **B** Box plot of Joint Line Height absolute difference by group raTKA vs mTKA. Mean difference in joint line height was significantly different (*p* = 0.027). The mean absolute value in joint line height difference between raTKA and mTKA groups was not significantly different (*p* = 0.235)

The raTKA and mTKA groups were comparable for age, sex and side distribution (Table 1). Excellent interobserver agreement was achieved for joint line and PCO measurements (ICC ≥ 0.9). The mean joint line height depression for the raTKA group was − 0.0001 mm (± 3.48, 95% CI − 0.509, 0.509), demonstrating an almost anatomical restoration of joint line height when calculating the postoperative to the preoperative difference in joint line height. The mean joint line height depression for the mTKA group was − 0.951 mm (± 4.33, 95% CI − 1.664, − 0.237), demonstrating a higher depression of the joint line postoperatively (Fig. 1A). The mean absolute values of joint line differences ([postoperative joint line height- preoperative joint line height]) for the raTKA group was 2.786 (± 2.08) mm, and for the mTKA group, 3.33 (± 2.75) mm (Fig. 1B). The comparison of the postoperative to the preoperative joint line for the raTKA group was not statistically significantly different (*p* = 0.523, Wilcoxon Signed Rank test). The comparison of the postoperative to the preoperative joint line, however, was significantly different for the mTKA group (*p* = 0.009, paired samples *t* test).

**Table 1:** Baseline demographics of the raTKA and mTKA groups

		mTKA group	raTKA group	*p* value
Age (years)^*^		70.51 (8.38)	70.54 (8.43)	0.847^@^
Sex^**^	Male	30 114	51 131	0.173^%^
	Female			
Side^**^	Right	73 71	83 99	0.423^%^
	Left			

*raTKA* robotically assisted total knee arthroplasty, *mTKA* manually performed total knee arthroplasty

*The values are given as the mean with the standard deviation ( ±) in parentheses

**The values are given as raw numbers

@Tests performed using the Mann–Whitney test

%Tests performed using the Chi-square (*x*^2^) test

**Fig. 2:**
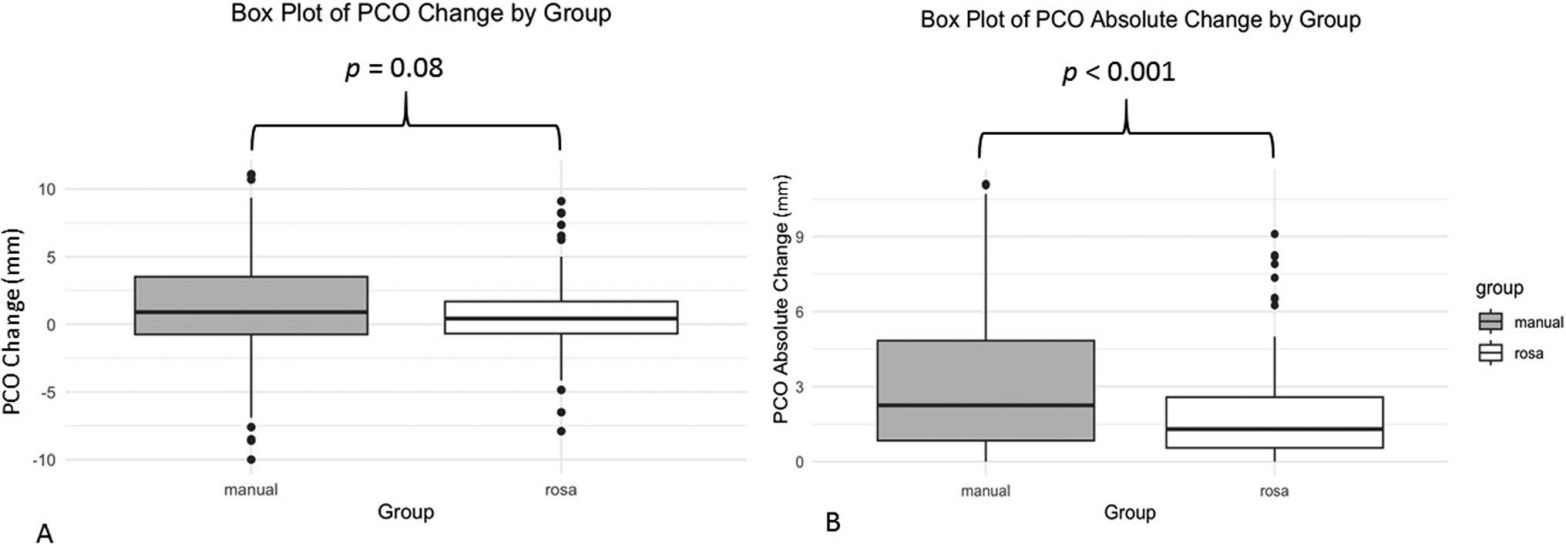
**A** Box plot of posterior condylar offset (PCO) average difference by group raTKA vs mTKA. **B** Box plot of PCO absolute difference by group raTKA vs mTKA. Mean difference in PCO, demonstrated a tendency towards significance (*p* = 0.08). The mean absolute value PCO difference between raTKA and mTKA groups was highly significant for PCO (*p* < 0.001)

The mean PCO difference for the raTKA group was 0.52 mm (± 2.45, 95% CI 0.160, 0.880), demonstrating a small mean postoperative PCO change. The mean PCO difference for the mTKA group was 1.15 mm (± 4.01, 95% CI − 1.496, 1.818), demonstrating a higher PCO change postoperatively (Fig. 2A). The mean absolute values of PCO differences ([postoperative PCO- preoperative PCO]) for the raTKA group was 1.799 (± 1.749) mm and for the mTKA group, 3.098 (± 2.79) mm (Fig. 2B). The comparison of the postoperative to preoperative PCO for the raTKA group was statistically significantly different (*p* = 0.005, paired samples *t* test). The comparison of the postoperative to preoperative PCO was also significantly different but with a higher statistical significance for the mTKA group (*p* < 0.001, paired samples *t* test).

The mean difference in joint line height of 0.95 mm between raTKA and mTKA groups was significantly different (independent samples t test, *p* = 0.027), and for PCO, it was 0.63 mm, which demonstrated a tendency towards significance (Mann–Whitney test, *p* = 0.08). The mean absolute difference in joint line height between raTKA and mTKA groups was not significantly different (Mann–Whitney test, *p* = 0.235) but highly significant for PCO (Mann–Whitney test, *p* < 0.001).

## Discussion

The restoration of the joint line as part of TKA is crucial as significant elevation can result in complications such as mid-flexion instability, patella mal-tracking and reduced ROM [1–3]. We have demonstrated ROSA robotic technology for TKA can restore joint line accurately to nearly anatomical and clearly better compared to the manual jig-based method. It can also better improve the restoration of PCO. Overall, it demonstrates the ability of robotic technology to achieve more precise positioning of the implant and may explain previous findings of improved patient satisfaction and Patient-Reported Outcome Measures (PROMs) [17].

The results are significantly better than what is reported to result in compromised functional outcomes. Early work by Figgie et al*.* (1986) demonstrated that less than 8 mm of joint elevation results in better functional outcomes in primary TKA [5]. More recently, a systematic review of studies of joint line alteration and postoperative outcome joint line elevation and postoperative KSS score were negatively correlated and achieved significance. For an ideal outcome joint line should be restored and the elevation should not exceed 4 mm [11]. A raised medial joint line results in mid-flexion laxity. When raised by 2 mm, there was an average of a 64% increase in mid-flexion laxity, and at 4 mm demonstrated a 111% increase. Normal joint laxity can be obtained when the joint line is corrected and can be demonstrated throughout the ROM arc [18]. In a more recent study with 120 patients who underwent primary TKA, the joint line position was assessed before and after surgery and demonstrated a rising trend in WOMAC score and a declining trend in KSS scores with every mm of joint line elevation. Elevation of the joint line ≥ 5 mm results in a poor outcome, and elevation of the joint line has damaging effects on outcome score after primary TKA [2]. Our outcomes demonstrated a mean joint line depression of 0.0001 mm and 0.951 mm for the raTKA and mTKA groups, respectively.

There have been different techniques used for the measurement of joint line. In a study undertaking measurements of 120 patients who underwent TKA and had joint line measurements the IJLCM method has excellent inter- and intra-rater reliability [13]. When measured from the tibial side, there was a significant difference in IKSS with the most difference at 24 months post-op [19]. In the current study, we also found excellent interobserver agreement for joint line and PCO measurements (ICC ≥ 0.9). Newer methods have been developed for the evaluation of patellar height and joint line position, for example, using the following parameters: ‘joint axis-patella (jAP), axis-patella (AP) and joint line height (JLH)’ as newly described by the authors as a way of assessing patellar height [20]. More recently, 3-D surface scans of the bone can be used and demonstrate high accuracy to ≤ 1 mm [21]. There is a requirement for further study to define which of the methods are superior.

### Posterior condylar offset

The effect of posterior condylar offset (PCO) on postoperative ROM in TKR has been of interest, as significant changes can result in reduced ROM. A postoperative decrease in PCO by more than 3 mm was can reduce ROM in cruciate retaining TKA [22]. We have demonstrated in our study that the use of ROSA robotic technology for TKA can more accurately restore PCO. PCO has been found to have an effect on the kinematics in cruciate sacrificing TKA, such as tibiofemoral translation in the posterior direction increasing as the PCO increases, albeit smaller than in CR KA due to the post-cam mechanism [23]. The functional outcome and long-term effects require further investigation.

### Benefits of using technology

Recently, there has been increasing use of robotic technology for the undertaking of TKA; however, studies investigating the effect on joint line are limited thus far. A recent investigation with patients undergoing robotic-assisted TKA using the NAVIO Surgical System had more accurate restoration of joint line and PCO compared to the jig-based technique [16]. Another study, which analysed 60 anteroposterior radiographs following the use of the NAVIO robotic technology system, was analysed. Measurements were taken from the joint line to the lateral femoral epicondyle (LEJL) and the proximal tibiofibular joint. Values subtracted from each other were found to be lower for the robotic group. This difference between the robotic and conventional manual groups showed a high statistical significance [12]. The ROSA robotic system is also an imageless robotic system, providing high accuracy with a joint line restoration nearly anatomical and evidence of improved restoration of PCO.

Limitations of the study include that all TKAs were carried out by the same surgeon and, therefore, the results may not be generalizable. There was also no randomization undertaken. The degree of correlation of the improved parameters with PROMs has not been analysed here. The improved degree of precision raTKA offers may be a vehicle for better PROMs but requires further correlational studies.

In conclusion, we have demonstrated that the ROSA robotic system can restore joint line position and PCO with greater accuracy than conventional manual jig-based techniques. The advantage of this study is the ability to compare with previous studies due to the use of the same measurement techniques, the accuracy and reliability of which have been previously demonstrated. Further study should correlate the accuracy of the joint line and PCO restoration to PROMs and the effects of the angle of the resections for the alignment strategy utilized.

## Data Availability

The data that support the findings of this study are available from the corresponding author upon reasonable request.
